# Statement of Retraction: Testis developmental related gene 1 promotes non-small-cell lung cancer through the microRNA-214-5p/Krüppel-like factor 5 axis

**DOI:** 10.1080/21655979.2024.2299553

**Published:** 2024-02-20

We, the Editors and Publisher of the journal *Bioengineered*, have retracted the following article:

Xudong Lu, Nian Zhao, Guangjun Duan, Zhiyong Deng & Yimin Lu (2022) Testis developmental related gene 1 promotes non-small-cell lung cancer through the microRNA-214-5p/Krüppel-like factor 5 axis, Bioengineered, 13:1, 603-616, DOI: 10.1080/21655979.2021.2012406

Since publication, significant concerns have been raised about the integrity of the data and reported results in the article. When approached for an explanation, the authors did not provide their original data or any necessary supporting information. As verifying the validity of published work is core to the integrity of the scholarly record, we are therefore retracting the article. All authors listed in this publication have been informed.

We have been informed in our decision-making by our policy on publishing ethics and integrity and the COPE guidelines on retractions.

The retracted article will remain online to maintain the scholarly record, but it will be digitally watermarked on each page as ‘Retracted’.

